# Sigmoid Colon Tuberculosis Revealed by a Perforation and Peritonitis

**DOI:** 10.7759/cureus.12272

**Published:** 2020-12-25

**Authors:** Hassane Ait Ali, Brahim Zeriouh, Rachid Jabi, Mohammed Bouziane

**Affiliations:** 1 General Surgery, Mohammed VI University Hospital Center, Oujda, MAR; 2 Oncology Surgery, Mohammed VI University Hospital Center, Oujda, MAR; 3 Visceral Surgery, Mohammed VI University Hospital Center, Oujda, MAR

**Keywords:** colon perforation, peritonitis, sigmoidectomy, sigmoid tuberculosis, surgical case reports

## Abstract

Intestinal tuberculosis is a frequent disease in developing countries, causing considerable morbidity and mortality. However, tuberculosis of the colon is rarer, and it also appears to be more common in immunosuppressed patients.

We report the case of a 71-year-old immunocompetent man who was admitted to the emergency department with an acute abdomen and features of perforation peritonitis. A sigmoid perforation on cancer was suspected on computed tomography (CT) scan and surgical exploration. A standard sigmoidectomy with end colostomy (Hartmann's procedure) and peritoneal toileting was done. The pathological assessment of the surgical specimen revealed the sigmoid colon tuberculosis, complicated by perforation and peritonitis. Thus, the unexpected diagnosis of sigmoid colon tuberculosis was only made after the histopathological examination. Then, he received anti-tuberculosis treatment for six months. Therefore, a complete colonoscopy was performed at the end of the treatment, which returned to be normal. Thereafter, the restoration of intestinal continuity was performed.

Colon tuberculosis is a rare disease and even rarer in people without immunodeficiency or on immunosuppressive therapy. If diverticulitis is the most common cause of sigmoid perforation, sigmoid perforation because of tuberculosis is extremely rare. However, an isolated primary sigmoid perforation of tubercular origin is not reported.

We report this exceptional case of sigmoid colon tuberculosis complicated by perforation and generalized peritonitis to sensitize the medical team to its rare occurrence, which will be of paramount importance due to the increasing incidence of tuberculosis worldwide.

## Introduction

Tuberculosis (TB) remains a worldwide health problem, especially in developing countries, with an estimated 9.0 million cases and 1.5 million deaths globally [1]. However, extrapulmonary tuberculosis affects an isolated organ or presents secondary to pulmonary involvement. Although the gastrointestinal tract is one of the sites most affected by tuberculosis [2], especially in the ileocecal area, a primary colonic manifestation without extraintestinal tuberculosis is exceptional. It is frequent in patients with acquired immune deficiency syndrome (AIDS), and those on immunosuppressive therapy.

Early diagnosis is difficult due to its nonspecific clinical presentation and its resemblance to malignancy or inflammatory bowel diseases. It includes abdominal pain, loss of weight and appetite, vomiting, and fever.

Intestinal tuberculosis is a treatable and curable disease by antituberculosis drugs, but surgery is only indicated for complications such as fistula, perforation, stricture, obstruction, or bleeding.

Hence, we report a case of intestinal tuberculosis located in the sigmoid colon in an immunocompetent man admitted for a colonic perforation and peritonitis, which is rare and almost indistinguishable from sigmoid colon cancer. The unexpected diagnosis was only made following histological examination of the surgical specimen.

We aim to raise awareness among the medical team of this rare pathology, which will be of paramount importance given the increasing incidence of tuberculosis in the world.

## Case presentation

A 71-year-old immunocompetent man was admitted to the emergency department with a week history of complaints of diffuse acute abdominal pain, fever, vomiting, abdominal distension, and constipation. On admission, he had no bowel motion and flatus for two days duration. In his surgical history, he was operated on for a left inguinal hernia four months ago and had no other significant prior medical or any evidence of a traumatic event. Additionally, he had no history of pulmonary or intestinal tuberculosis and no immunosuppressive therapy. His family history was unremarkable.

Physical examination showed a blood pressure of 120/60 mmHg, a regular pulse at 84/min, and a body temperature of 38.6°C. The patient's abdomen was distended, and he had diffuse abdominal tenderness, especially at the left iliac fossa with intense signs of peritoneal irritation. The peristaltic sounds were not audible. A digital rectal exam (DRE) found an empty rectal vault.

Laboratory studies (Table 1) revealed a hemoglobin concentration of 15.3 g/dL, a white blood cell (WBC) count of 15,200 cells/μL with 84.3% neutrophils, a platelet count of 390,000 cells/μL and a high C-reactive protein (CRP) level of 128 mg/dl. Renal and liver function tests were normal. Serum carcinoembryonic antigen (CEA) and carbohydrate antigen 19-9 (CA 19-9) levels were normal.

**Table 1:** Laboratory data for blood on admission GOT: Glutamic Oxaloacetic Transaminase; GPT: Glutamic Pyruvic Transaminase; LDH: Lactate Dehydrogenase; Gamma-GT: Gamma-Glutamyl Transferase; CRP: C-reactive Protein; AFP: Alpha Fetoprotein; CEA: Carcinoembryonic Antigen.

WBC (white blood cells)	15,200/μl
Neutrophils	12,810/μl (84.3%)
Lymphocytes	1170/μl (7.7%)
Monocytes	1170/μl (7.7%)
Eosinophils	10/μl (0.06%)
Basophils	40/μl (0.26%)
RBC (Red Blood Cells)	4.5 x 10^6^/μl
Hemoglobin	15.3 g/dl
Hematocrit	43.2%
Platelets	390,000/μl
Total protein	70 g/l
Albumin	42 g/l
GOT	14 U/l
GPT	13 U/l
LDH	164 U/l
Gamma-GT	27 U/l
Total bilirubin	12 mg/l
CRP	128 mg/dl
Creatinine	7.54 mg/l
Urea	0.42 g/l
Na 135	136 mEq/l
K 4.3	5 mEq/l
AFP	1.7 ng/ml
CA 19-9	12 U/ml
CEA	3 ng/ml
PT (prothrombin time)	86%

**Figure 1:**
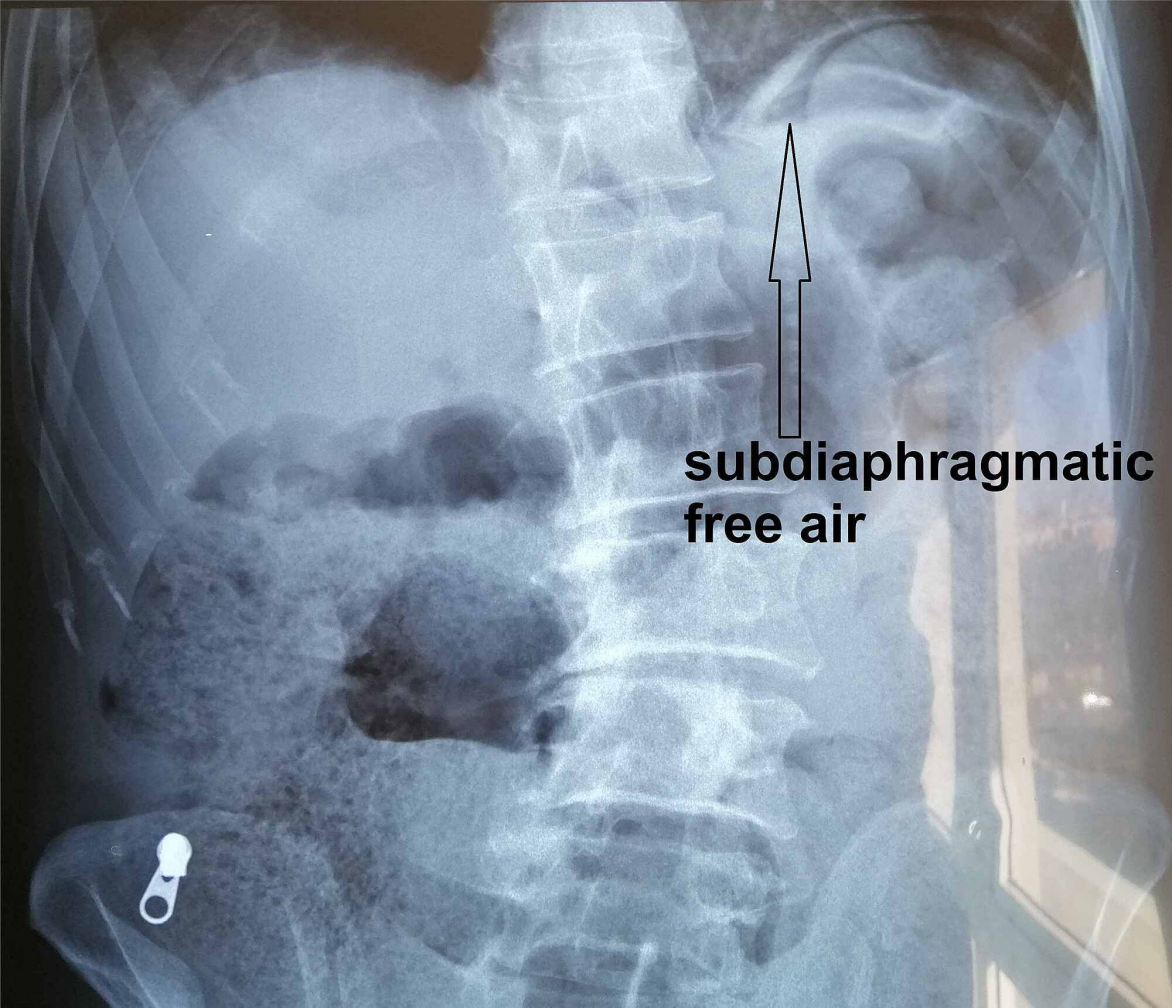
X-ray of abdomen X-ray of abdomen, showing sub-diaphragmatic free air.

Subdiaphragmatic free air was detected on plain chest X-ray study (Figure 1).

**Figure 2:**
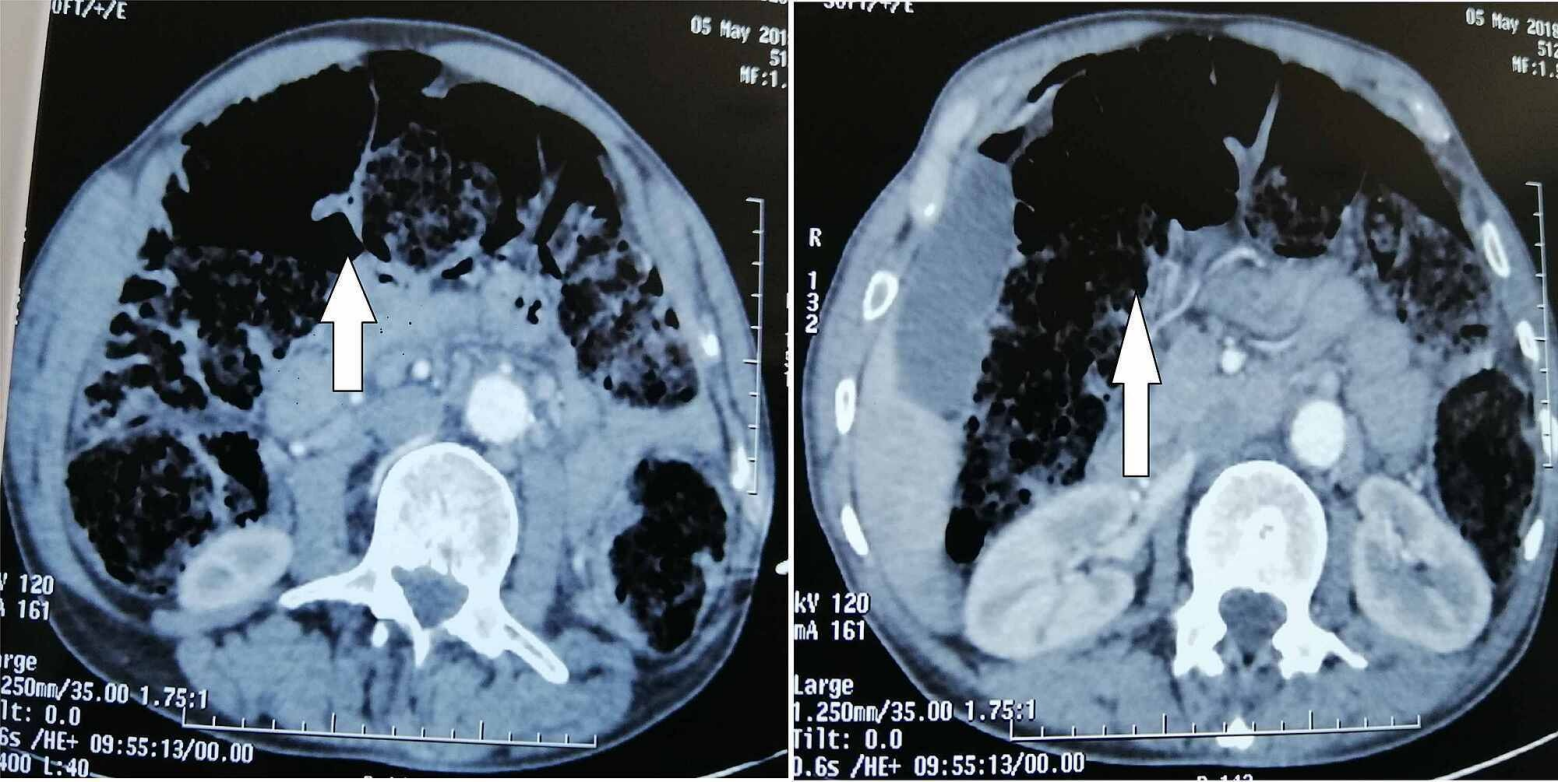
Abdominal CT scan Axial CT scan showing a distention of small and large bowel (White arrow).

**Figure 3:**
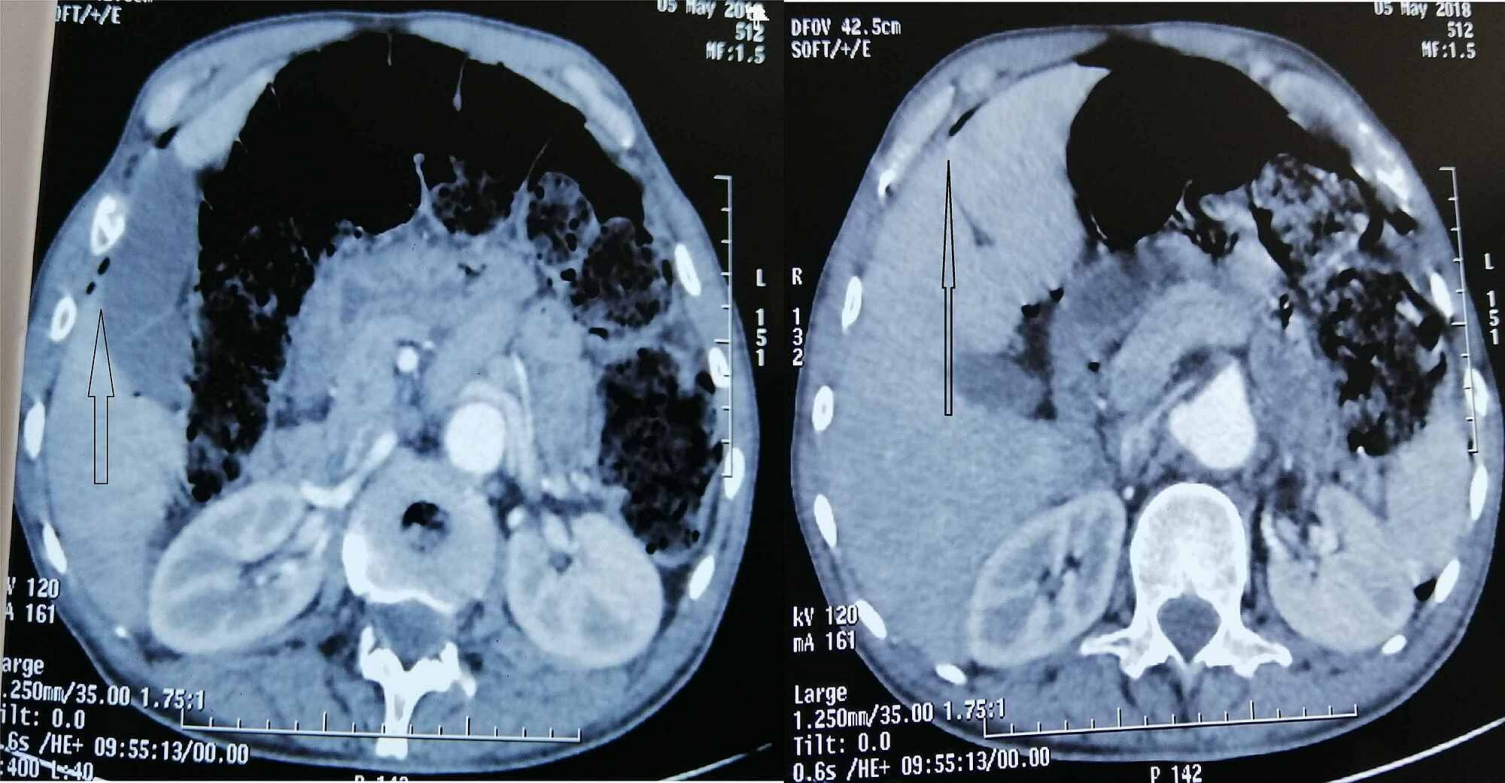
Abdominal CT scan Axial CT scan of the patient on admission showing free air in the peritoneal cavity.

An abdominal CT scan (Figures 2, 3) revealed air in the peritoneal cavity, free fluid in the pelvis, and a perforated thickened intestinal wall located in the sigmoid colon, which occludes the lumen, responsible for upstream intestinal obstruction. There were no lesions within the liver.

After a short preparation and hydroelectrolytic management, the patient was taken up for an emergency laparotomy. During the exploration, intra-abdominal purulent fluid, pseudomembranes, and a perforated mass of the sigmoid colon were observed. The rest of the bowel appeared completely normal. The liver and spleen were normal, and there was no lymphadenopathy. Thus, the perforated mass in the sigmoid colon (SC) was suspected as a malignant lesion.

The management strategy consisted of a two-stage approach comprised of initial sigmoid resection and end colostomy (Hartmann's procedure) followed by a second surgery to restore intestinal continuity. It is associated with regional lymphadenectomy, including ligation of the lower mesenteric artery and vein.

We did not perform a primary intestinal anastomosis because there was diffuse peritonitis; failure of the anastomosis was probable.

During his stay at the hospital, a prophylaxis for thromboembolism, analgesia, and ceftriaxone (2g IV per day) with metronidazole 500mg IV (500 mg every 8 hours) antibiotics were administered. The early postoperative period was uneventful, and the patient improved rapidly. He resumed oral intake on the first day postoperatively, and his first defecation from colostomy was at two days postoperatively. The patient was discharged from the hospital after five days.

Microscopic examination revealed multiple submucosal granulomas, with gigantic cell aggregations and localized granuloma necrosis, which is also compatible with colonic tuberculosis.

Following the result of the histological examination, we performed a retrospective analysis of our patient. The X-ray chest was normal, quantiferon gold was negative, and sputum was negative (three samples). Although, the patient had no other sites of tuberculosis infection like pulmonary or urinary.

The patient received a standard tuberculosis treatment for six months, which included ethambutol, rifampicin, isoniazid, with pyrazinamide for the first two months, and then rifampicin, and isoniazid for the next four months, with satisfactory results. Therefore, four weeks after the end of the treatment, a complete colonoscopy was performed, which was negative for M. tuberculosis. Then the colostomy closure is done in the second operation by a midline laparotomy.

The patient’s postoperative course was uneventful, and bowel transit for gas and feces returned after three days. Then, the patient was discharged from the hospital in five days.

He was regularly followed-up. He was free of recurrence and was in good health for four years after surgery.

## Discussion

Tuberculosis (TB) is an infectious disease caused by the bacillus Mycobacterium tuberculosis. It typically affects the lungs (pulmonary TB) but can also affect other sites (called extrapulmonary tuberculosis). It occurs in every part of the world, and over 95% of cases and deaths are in developing countries. In Morocco, tuberculosis is one of the most frequent diseases, with more than 30,000 people affected every year.

Extra-pulmonary tuberculosis affects an isolated organ or presents secondary to pulmonary involvement. It accounts for 11-16% of all tuberculosis, and abdominal location represents only 3 to 4% [3]. However, colonic tuberculosis represents 12.1% of all gastrointestinal tuberculosis cases and only 6% of all primary intra-abdominal localizations [4]. The location intestinal frequently occurs in adults with a ratio of approximately 1:2. Generally, the infection is caused by swallowed infected sputum (from pulmonary infection), hematogenous and contiguous spread from adjacent organs, or contaminated food.

On the other hand, the aging of society, the prevalence of HIV, diabetes, chronic renal failure, and the increasing use of immunosuppressants, including anti-tumor necrosis factor-alpha (TNF-a), could increase tuberculosis infection. However, our 71-year-old male patient had neither HIV infection nor extraintestinal tuberculosis but was at increased risk because of his origin and age. As Morocco is considered an endemic area for tuberculosis, reactivation of latent infection should be considered a possible cause, even in an immunocompetent host.

Additionally, the location frequently affected is in the ileocecal area, followed by the ascending colon, jejunum, appendix, duodenum, stomach, sigmoid colon, and rectum [5]. The frequent occurrence in the ileocecal area is due to the physiological stasis and the abundance of lymphatic tissues [6]. As a result, sigmoid colon tuberculosis is exceptional and can be misdiagnosed as: colon cancer, inflammatory bowel diseases, ischemic colitis, or infectious colitis. To our knowledge, a few previous studies have reported on tuberculosis that occurs in the sigmoid colon.

The diagnosis is difficult and often delayed due to its non-specific presentation. However, most patients are diagnosed after exploratory laparotomy with bowel resection. Furthermore, the most common presenting symptoms were pain and vomiting. Additionally, they can range from only mild abdominal pain to intestinal obstruction of the lumen of the gut or even perforation [7], whereas gastrointestinal bleeding was rare [8]. Other complications have been reported, such as Fistula occurring at different sites, stricture formation, and intussusception. However, complications can be the first clinical presentation of the disease, as proven by our clinical case. Usually, sigmoid perforations are seen as associated with diverticular disease [9], but sigmoid perforation because of tuberculosis is exceptional. Until today, a few cases of colonic perforation from TB have been reported in the literature, and these patients presented with peritonitis [10], but sigmoid colon perforation because of tuberculosis is extremely rare.

In our case, tuberculosis was proven by the pathological examination of the surgical specimen, which was considered from sigmoid in origin. To our knowledge, this report is the first case of sigmoid tuberculosis complicated by perforation and generalized peritonitis.

The most common endoscopic presentations are intestinal inflammation, with four major forms have been described [11]:

Ulcerative - the most common form. Usually presents with superficial transverse ulcers. It is more likely to be seen in the small intestine.

Hypertrophic - occurs as a hyperplastic reaction around the ulcer, producing an inflammatory mass. It is more likely to be seen in the cecum.

Ulcero-hypertrophic - a combination of ulcerative and hypertrophic forms may occur.

Fibrous stricturing - may lead to fibrosis and stricture formation, resulting in intestinal obstruction.

As in this case, the confirmatory diagnosis is made after the histological examination. The histopathology findings, either from colonoscopy or from colectomy of the affected colon, include the image of chronic colitis with ulceration of the mucosa, the growth of granulomas, and lymph node infiltration.

The clinical and radiologic features of intestinal tuberculosis may mimic many diseases and it is usually called the great mimicker. Therefore, the differential diagnosis includes: Crohn's disease, amebiasis, colorectal cancer, Yersinia enterocolitis, gastrointestinal histoplasmosis, and peri-appendiceal abscesses closely simulate intestinal tuberculosis [12].

Regarding the treatment of intestinal tuberculosis, most current guidelines recommend treating with antituberculous treatment (ATT) for six months. However, some clinicians treat for longer periods due to concerns that six months is not adequate to achieve a cure and prevent relapse of the disease. A standard four-drug regimen, which includes isoniazid (H), rifampicin (R), and pyrazinamide (Z), usually with ethambutol (E) as a fourth drug during the first two months of treatment (intensive phase), followed by isoniazid and rifampicin for four additional months (continuation phase) [13,14]. However, surgery may be needed in the setting of complications such as obstruction, perforation, and fistulation or bleeding [15].

In the current case, our primary impression was a sigmoid colon tumor. Nevertheless, the pathological examination redressed the diagnosis, and anti-tuberculosis treatments were prescribed subsequently for six months.

On the other hand, Hartmann's procedure is the preferred option over primary anastomosis for sigmoid perforation with purulent or fecal peritonitis.

Colonoscopy is important for follow-up, which shows either healing of previously seen mucosal lesions or mild mucosal irregularities [16]. It should be conducted two to three months after anti-tuberculosis medication. In our case, we performed a follow-up colonoscopy one month after the end of treatment to search for other tuberculosis sites and to exclude recurrence before restoration of digestive continuity. Therefore, it revealed no anomalies, and a restoration of continuity is performed by Medline laparotomy.

## Conclusions

Intestinal and peritoneal tuberculosis is highly prevalent in developing countries. Although, any portion of the gastrointestinal tract may be affected. However, a primary isolated sigmoid perforation due to tuberculosis is an unusual occurrence.

In this clinical presentation, we reported an exceptional case of sigmoid colon tuberculosis complicated by perforation and peritonitis, which is successfully treated by surgery and anti-tuberculosis medication. The aims and objective of this case are to raise awareness among the medical team about tuberculosis again. Nevertheless, its incidence rates are increasing, and it will certainly take a more important role in the future. Therefore, it should be sought in patients with abdominal pain and an increased risk of tuberculosis or those with proven pulmonary tuberculosis.
